# O Coração e a COVID-19: O que o Cardiologista Precisa Saber

**DOI:** 10.36660/abc.20200279

**Published:** 2020-05-22

**Authors:** Isabela Bispo Santos da Silva Costa, Cristina Salvadori Bittar, Stephanie Itala Rizk, Antônio Everaldo de Araújo, Karen Alcântara Queiroz Santos, Theuran Inahja Vicente Machado, Fernanda Thereza de Almeida Andrade, Thalita Barbosa González, Andrea Nataly Galarza Arévalo, Juliano Pinheiro de Almeida, Fernando Bacal, Gláucia Maria Moraes de Oliveira, Marcus Vinícius Guimarães de Lacerda, Silvio Henrique Barberato, Antonio Carlos Palandri Chagas, Carlos Eduardo Rochitte, José Antonio Franchini Ramires, Roberto Kalil, Ludhmila Abrahão Hajjar

**Affiliations:** 1 Universidade de São Paulo Instituto do Câncer do Estado de São Paulo São PauloSP Brasil Universidade de São Paulo - Instituto do Câncer do Estado de São Paulo, São Paulo, SP - Brasil; 2 Universidade de São Paulo Instituto do Coração São PauloSP Brasil Universidade de São Paulo Instituto do Coração, São Paulo, SP - Brasil; 3 Universidade Federal do Rio de Janeiro Rio de JaneirRJ Brasil Universidade Federal do Rio de Janeiro – Cardiologia, Rio de Janeiro, RJ - Brasil; 4 Fundação de Medicina Tropical Doutor Heitor Vieira Dourado ManausAM Brasil Fundação de Medicina Tropical Doutor Heitor Vieira Dourado, Manaus, AM - Brasil; 5 CardioEco Centro de Diagnóstico Cardiovascular CuritibaPR Brasil CardioEco Centro de Diagnóstico Cardiovascular, Curitiba, PR - Brasil; 6 Quanta Diagnóstico CuritibaPR Brasil Quanta Diagnóstico – Ecocardiografia, Curitiba, PR - Brasil; 7 Faculdade de Medicina Fundação do ABC Santo AndréSP Brasil Faculdade de Medicina da Fundação do ABC, Santo André, SP - Brasil; 8 Hospital do Coração São PauloSP Brasil Hospital do Coração, São Paulo, SP – Brasil

**Keywords:** Coronavirus, COVID-a9, Doenças Cardiovasculares/complicações, Coração/fisiopatologia, Pandemias, Síndrome de Desconforto Respiratório do Adulto, Fatores de Risco, Assistência ao Paciente

## Abstract

Frente à pandemia da doença causada pelo novo coronavírus (COVID-19), o manejo do paciente com fator de risco e/ou doença cardiovascular é desafiador nos dias de hoje. As complicações cardiovasculares evidenciadas nos pacientes com COVID-19 resultam de vários mecanismos, que vão desde lesão direta pelo vírus até complicações secundárias à resposta inflamatória e trombótica desencadeada pela infecção. O cuidado adequado do paciente com COVID-19 exige atenção ao sistema cardiovascular em busca de melhores desfechos.

## Introdução

O mundo hoje vive a pandemia da doença causada pelo novo coronavírus (COVID-19), afecção que surgiu em dezembro de 2019 na cidade de Wuhan, província de Hubei, na China.^1 , 2^ Os casos iniciais foram descritos como pneumonia de rápida evolução para síndrome do desconforto respiratório agudo (SDRA).

Esse novo vírus, denominado SARS-CoV-2 (do inglês: *severe acute respiratory syndrome coronavirus 2* ), é o sétimo coronavírus identificado até o momento e difere dos outros coronavírus que causam resfriado comum e pneumonia leve (229E, OC43, NL63 e HKU1). O SARS-CoV-2 assemelha-se aos vírus da síndrome aguda respiratória grave por coronavírus (SARS) e da síndrome aguda respiratória grave do Oriente Médio (MERS), ocorridas na China em 2002-2003 e no Oriente Médio em 2012, respectivamente.^1 , 2^ Embora existam semelhanças filogenéticas entre o SARS-CoV-2 e os coronavírus zoonóticos que causaram a SARS e a MERS, a transmissibilidade do SARS-CoV-2 é muito maior, contribuindo para uma disseminação da infecção até dez vezes mais rápida que a do SARS-CoV.^2 - 4^ O número básico de reprodução (R0) da doença é de 2,78, ou seja, cada indivíduo infectado tem a capacidade de transmitir a doença para em média 3 pessoas.^5^ Em estudo recente publicado na revista *Science* , em modelo matemático, postulou-se que cerca de 85% das transmissões da COVID-19 ocorram por indivíduos assintomáticos.^6^

Em decorrência da rápida disseminação, a COVID-19 foi declarada uma pandemia pela Organização Mundial da Saúde (OMS) em 11 de março de 2020.^4^ Atualmente, a COVID-19 afeta mais de 181 países em todo o mundo e o número de casos cresce de forma exponencial. Até 2 de abril de 2020, havia um total de 1.015.403 casos registrados globalmente e 53.030 mortes, o que resulta em uma taxa de letalidade de 5,2%. Na mesma data, o Brasil computava 8.044 casos confirmados e 324 óbitos, com mortalidade de 4%. Dados brasileiros iniciais mostram que 90% dos óbitos ocorreram em pessoas com idade maior de 60 anos e 84% dos pacientes apresentavam pelo menos uma comorbidade, sendo que 51% tinham doença cardiovascular (DCV) e 37,7% tinham diabetes.^7^

A análise de 44.672 casos confirmados de COVID-19 em Wuhan evidenciou uma taxa de letalidade geral de 2,3%. Porém, a letalidade foi maior em DCV (10,5%), diabetes (7,3%) e hipertensão arterial (6%).^8^ Também foram descritas complicações cardiovasculares decorrentes da COVID-19, como injúria miocárdica (20% dos casos), arritmias (16%), miocardite (10%), além de insuficiência cardíaca (IC) e choque (até 5% dos casos).^9 - 11^

O objetivo desta revisão é auxiliar o clínico, o emergencista, o cardiologista e o intensivista na assistência aos pacientes com COVID-19, propondo um algoritmo de avaliação cardiovascular para a detecção precoce de complicações, além de recomendar protocolos de tratamento de complicações cardiovasculares nesses pacientes.

### Consequências da COVID-19 no sistema cardiovascular

**Figura 1:**
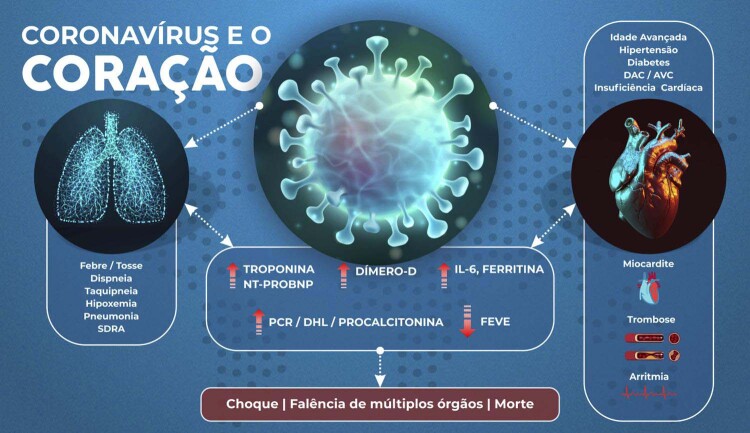
Coronavírus e o Coração. Os pacientes com fatores de risco e/ou doença cardiovascular são mais propensos a desenvolver formas graves e complicações relacionadas a COVID-19. O quadro pulmonar manifesta-se inicialmente por síndrome gripal (com tosse e febre), evolui para pneumonia (dispneia, hipoxemia, taquipneia) e, em alguns casos, para síndrome do desconforto respiratório agudo. A resposta do organismo ao vírus leva a um quadro de inflamação sistêmica, na qual se observa elevação de marcadores inflamatórios (PCR, procalcitonina, dímero-d, IL-6, ferritina, DHL) e de injúria miocárdica / disfunção cardíaca (troponina/NT-proBNP), que predispõe a insuficiência cardíaca aguda, miocardite, trombose e arritmias. As complicações cardiovasculares pioram a resposta do organismo ao vírus, levando a choque, falência de múltiplos órgãos e morte. AVC: acidente vascular cerebral; DAC: doença arterial coronária; DHL: desidrogenase láctica; FEVE: fração de ejeção do ventrículo esquerdo; PCR: proteína C reativa; IL-6: interleucina-6; SDRA: síndrome do desconforto respiratório agudo.

Dados recentes da pandemia da COVID-19 descrevem que o vírus pode afetar o sistema cardiovascular com manifestações diversas como injúria miocárdica, IC, síndrome de Takotsubo (ST), arritmias, miocardite e choque.^4 , 11 - 14^ O dano ao sistema cardiovascular é provavelmente multifatorial e pode resultar tanto de um desequilíbrio entre alta demanda metabólica e baixa reserva cardíaca quanto de inflamação sistêmica e trombogênese, podendo ainda ocorrer por lesão direta cardíaca pelo vírus.^13^ Esse dano ao sistema cardiovascular decorrente da COVID-19 ocorre principalmente nos pacientes com fatores de risco cardiovascular (idade avançada, hipertensão e diabetes) ou com DCV prévia.^10 , 11^ A Figura 1 sumariza a resposta inflamatória gerada a partir da infecção viral que leva à lesão do sistema cardiovascular e dos pulmões, com elevação de dímero-D, procalcitonina, proteína C reativa, ferritina, troponina e NT-proBNP, e que culmina em complicações cardiovasculares e óbito.

Na resposta inflamatória sistêmica provocada pela COVID-19, observam-se concentrações mais altas de citocinas que estão relacionadas à injúria do sistema cardiovascular.^15^ O aumento de troponina é acompanhado de elevação de outros marcadores inflamatórios, como dímero-D, ferritina, interleucina-6 (IL-6), desidrogenase láctica (DHL), proteína C reativa, procalcitonina e contagem de leucócitos.^1 , 11^ Zhou et al. mostraram que os pacientes que evoluíram a óbito apresentaram níveis mais altos de dímero-D, IL-6, ferritina e DHL, além de linfopenia, sugerindo que esses marcadores inflamatórios possam ter implicações prognósticas. Dímero-D na admissão maior que 1µg/ml foi preditor independente de mortalidade nessa população.^12^ Além da elevação dos marcadores inflamatórios, nos pacientes com COVID-19 também se observa aumento nos níveis de BNP ou NT-proBNP, marcadores de disfunção miocárdica. Pacientes com injúria miocárdica tiveram níveis mais altos de NT-proBNP, com correlação linear positiva.^10 , 11^ Esse achado reforça que aqueles que apresentam injúria miocárdica estão mais propensos a desenvolver comprometimento da função cardíaca.^10^

Numa meta-análise de quatro estudos, incluindo um total de 341 pacientes, os níveis de troponina I foram significativamente maiores naqueles com a forma grave da COVID-19 em comparação àqueles com a forma não grave.^16^ Os pacientes com injúria miocárdica internaram mais em unidade de terapia intensiva (UTI) (22,2% vs. 2,0%), tiveram maior incidência de IC (52% vs 12%) e maior taxa de óbito (59% vs. 1%).^1 , 9^ Shi et al. avaliaram 416 pacientes internados por COVID-19 e observaram que a injúria miocárdica, definida pela elevação dos níveis de troponina maior que o percentil 99 do valor de referência, é complicação frequente (19,7%) nesses pacientes e está associada com aumento de mortalidade e SDRA.^11^ No modelo de análise multivariada, injúria miocárdica e SDRA foram preditores independente de mortalidade (HR de 4,26 e 7,89, respectivamente).^11^ Em estudo recente publicado por Guo et al., 27,8% de 187 pacientes apresentavam elevação de troponina. A mortalidade foi de 7,6% em pacientes sem DCV e com níveis normais de troponina, de 13,3% em pacientes com DCV e troponina normal, de 37,5% em pacientes sem DCV e troponina elevada, e de 69,4% em pacientes com DCV e troponina alterada. Houve forte correlação entre níveis altos de troponina e aumento de proteína C reativa e de NT-proBNP. Pacientes com níveis aumentados de troponina tiveram maior incidência de arritmias ventriculares e maior necessidade de ventilação mecânica.^10^

Complicações cardiovasculares, como IC, miocardite, infarto agudo do miocárdico, choque e arritmias, também são frequentes em pacientes com injúria miocárdica. Em uma coorte com 150 pacientes, 7% deles desenvolveram dano miocárdio e IC irreversíveis, associados a elevações significativas dos níveis de troponina.^17^ Arritmias malignas (taquicardia ventricular com degeneração para fibrilação ventricular ou instabilidade hemodinâmica) foram observadas com maior frequência nos grupos com elevação dos níveis de troponina (11,5% vs 5,2%).^10^ Pacientes com COVID-19, quando apresentam a forma grave da doença, podem evoluir rapidamente para quadro com importante comprometimento cardiovascular, choque e falência múltipla de órgãos. Nas coortes chinesas com pacientes internados, até 20% evoluíram para quadros graves com choque.^9 , 12^

Miocardite pode estar relacionada a falência cardíaca aguda nos pacientes com COVID-19. Foram descritos casos de miocardite relacionada à COVID-19, como miocardite fulminante, de rápida evolução e disfunção ventricular importante, associada a edema miocárdico difuso. Alterações eletrocardiográficas e elevação de troponina estavam presentes nesses pacientes.^14 , 18 , 19^ Apesar de não haver relato de ST diretamente relacionada à COVID-19, postula-se que alguns casos de disfunção ventricular nesses pacientes possam decorrer dessa síndrome. A ST é complicação frequente em indivíduos com resposta inflamatória sistêmica exacerbada, funcionando o estresse e a gravidade da infecção viral como gatilho para a ST.^20^

### Interação do SARS-CoV-2 com a enzima de conversão da angiotensina 2

**Figura 2:**
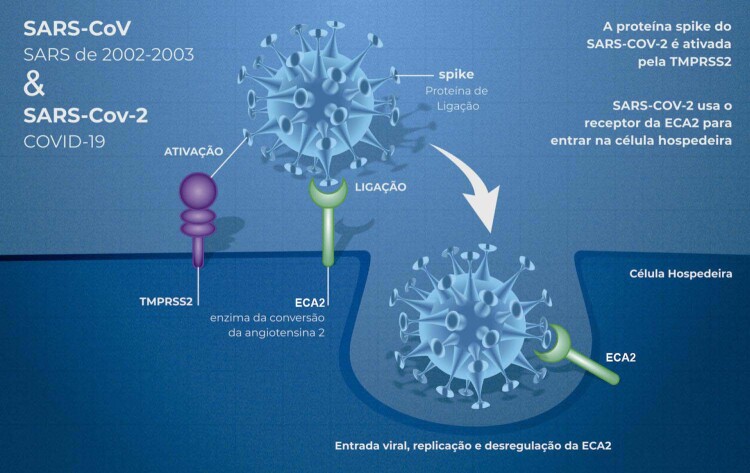
O SARS-CoV-2 liga-se por meio da proteína spike da superfície viral ao receptor da ECA2 humana após a ativação da proteína spike pela TMPRSS2.SARS-CoV: Coronavírus da síndrome respiratória aguda grave; SARS-COV-2: Coronavírus da síndrome respiratória aguda grave 2; COVID-19: doença do coronavírus 2019; ECA2,:enzima conversora de angiotensina-2; TMPRSS2: serina protease transmembrana-2.

Alguns estudos sugerem que a lesão ao sistema cardiovascular secundária ao vírus possa estar relacionada à enzima de conversão da angiotensina 2 (ECA2).^13 , 15^ A ECA2 está relacionada ao sistema imune e presente em alta concentração no pulmão e no coração. A ECA2 regula negativamente o sistema renina angiotensina pela inativação da angiotensina-2 e provavelmente tem um papel protetor contra o desenvolvimento de insuficiência respiratória e sua progressão. O SARS-CoV-2 contém quatro proteínas estruturais principais: a proteína *spike* (S), a proteína nucleocapsídeo (N), a proteína membrana (M) e o envelope proteico (E). O vírus liga-se por meio da proteína *spike* ao receptor da ECA2 e, por meio dessa ligação, entra na célula hospedeira ( Figura 2 ), onde ocorre a inativação da ECA2, o que favorece a lesão pulmonar. Como a ECA2 apresenta concentrações elevadas no coração, lesões potencialmente graves ao sistema cardiovascular podem ocorrer.^13 , 21^

Pacientes com DCV preexistente parecem ter níveis séricos aumentados da ECA2, o que pode contribuir para as manifestações mais graves nessa população.^22 - 24^ Da mesma forma, indivíduos com hipertensão arterial apresentariam maior expressão da ECA2 secundária ao uso de inibidores da enzima de conversão da angiotensina (IECA) ou bloqueadores do receptor da angiotensina II (BRA), o que potencialmente aumentaria a susceptibilidade à infecção pelo SARS-CoV-2.^4^ Entretanto, os estudos atuais em humanos apresentaram limitações: a) avaliaram um número pequeno de indivíduos em uso dessas medicações, e b) grande parte dos analisados era de idosos, fator confundidor importante, uma vez que a idade avançada aumenta a susceptibilidade à infecção, além de esse ser o principal fator de mau prognóstico.^25^

Outro ponto a ser considerado é que, apesar de a ECA2 e a ECA serem enzimas com estruturas homólogas, os sítios de ativação são distintos e, dessa forma, a inibição da ECA não teria efeito direto sobre a atividade da ECA2; essa enzima tem papel bem reconhecido na recuperação da função ventricular em pacientes com lesão miocárdica, por sua inibição da atividade da angiotensina II.^26^ Por outro lado, sugere-se que a angiotensina II seja responsável pelo dano cardíaco do coronavírus e a administração de ECA2 recombinante normalizaria os níveis de angiotensina II. Estudos estão sendo realizados com ECA2 recombinante e com losartana.^25^

A recomendação atual é que os IECA e os BRA sejam mantidos nos pacientes que já estão em uso regular dessas medicações pelo claro benefício do controle pressóricos e da diminuição de mortalidade naqueles com IC, como evidenciado em estudos randomizados.^27 , 28^ Nas formas graves da COVID-19, deve-se avaliar individualmente a estabilidade hemodinâmica e a função renal antes de decidir pela manutenção ou suspensão das medicações.

### A doença cardiovascular como grupo de risco para forma grave de COVID-19

Pacientes com fatores de risco cardiovasculares (idade avançada, hipertensão e diabetes), assim como aqueles com DCV (doença arterial coronária, cardiomiopatias e doença cerebrovascular) estão suscetíveis a desenvolver a forma grave da doença e complicações cardiovasculares, sendo classificados como grupo de risco. Aproximadamente 80% dos pacientes com a forma grave da doença têm alguma comorbidade.^29^ A Tabela 1 apresenta um resumo dos principais estudos que caracterizam as comorbidades clínicas dos pacientes com COVID-19.^9 - 12 , 17 , 29 - 32^

**Tabela 1:** Resumo das características clínicas dos principais estudos sobre COVID-19

Autor	N	Tipo	Idade (anos)	Comorbidades	Principais achados
Huang et al. 2020^17^	41	Prospectivo	49 (41-58)	- DM: 8 (20%) - HAS: 6 (15%) - DCV: 6 (15%) - DPOC: 1 (2%) - Câncer: 1 (2%)	- 13 (32%) internação em UTI - 5 (12%) IM, sendo que 4 (31%) foram para UTI - 3 (7%) choque e 12 (29%) SDRA - Mortalidade: 6 (15%)
Wang et al. 2020^30^	69	Retrospectivo	42 (35-62)	- HAS: 9 (13%) - DCV: 8 (12%) - DM: 7 (10%) - DPOC: 4 (6%) - Câncer: 4 (6%)	- Hospitalização: 44 (65.7%) - Mortalidade: 5 (7,5%) - Pacientes com DM, HAS e DCV apresentavam mais hipoxemia (SatO_2_ < 90%) - Não avaliado IM
Chen et al. 2020^31^	99	Retrospectivo	55 (21-82)	- DCV: 40 (40%) - DM: 12 (12%) - Câncer: 1 (1%)	- 57 (58%) hospitalização, 17 (17%) SDRA, 4 (4%) choque - Mortalidade: 11 (11%) - Dos óbitos, 63% tinham > 60 anos e 33% HAS
Wang et al. 2020^9^	138	Retrospectivo	56 (42-68)	- HAS: 43 (31,2%) - DCV: 20 (14,5%) - DM: 14 (10,1%) - Câncer: 10 (7,2%) - AVC: 7 (5,1%)	- 36 (26%) internação em UTI, prevalência elevada de fatores de risco - 12 (8,7%) choque, 23 (16,7%) arritmias, 27 (19,6%) SDRA e 10 (7,2%) IM - Mortalidade: 6 (4,3%)
Zhang et al. 2020^29^	140	Retrospectivo	57 (20–83)	- HAS: 42 (30%) - DM: 17 (12,1%) - DAC: 7 (5%) - Arritmias:5(3,6%)	- Comparando grupo grave x não grave: mediana idade 64 vs 51.5, p < 0,001 comorbidades 79,3% vs 53,7%, p =0,002 dímero-D 0,4 vs 0,2, p<0,001
Guo et al. 2020^10^	187	Retrospectivo	58,5 (±14,7)	- HAS: 61 (32,6%) - DAC: 21 (11,2%) - IC: 8 (4,3%) - DM: 28 (15%) - DPOC: 4 (2,1%) - Câncer: 13 (7%)	- 52 (27,8%) IM - Comparando tropo nl x tropo elevada: HAS 27% vs 63,5%, p 0.001 DAC 3% vs 32,7%, p <0.001 IC 0% vs 15,4%, p <0,001 - 43 mortes, sendo 31 (59,6%) no grupo IM - Mortalidade: 13,3% DCV sem IM e 69,4% DCV com IM
Zhou et al 2020^12^	191	Retrospectivo	56 (46-67)	- HAS: 58 (30%) - DM: 36 (19%) - DAC: 15 (8%) - DPOC: 6 (3%) - Câncer: 2(1%)	- IM: 24/145 (17%), mais elevada em pacientes que evoluíram a óbito (22,2 [5,6-83,1] vs 3,0 [1,1-5,5], p <0,001) - IC 44 (23%), choque 38 (20%), SDRA 59 (31%) - 54 (28%) óbitos, 67% com comorbidades
Shi et al. 2020^11^	416	Prospectivo	64 (21-95)	- HAS: 127 (30.5%) - DM: 60 (14,4%) - DAC: 44 (10,6%) - AVC: 22 (5,3%) - IC: 17 (4,1%) - Câncer: 9 (2,2%)	- 82 (19,7%) IM - Prevalência alta de HAS, DM, DAC e IC nos pacientes com IM - IM esteve relacionada com maior mortalidade: (42 de 82 [51,2%] vs 15 de 334 [4,5%]; p < 0,001) - IM esteve associada com SDRA: (48 de 82 [58,5%] vs 49 de 334 [14,7%]; p < 0,001)
Guan et al. 2020^32^	1099	Retrospectivo	47 (35-58)	- DPOC: 12 (1,1%) - DM: 81 (7,4%) - HAS: 165 (15%) - DAC: 27 (2,5%) - AVC: 15 (1,4%) - Câncer: 10 (0,9%)	- Os pacientes graves: HAS 41 (23,7%) - Elevação de CK-MB 90/657 (13,7%) - 12 (1,1%) choque, 37 (3,4%) SDRA, 1029 (93,6%) hospitalizações, 55 (5%) admissão em UTI - Mortalidade: 15 (1,4%)

*DM: diabetes mellitus; HAS: hipertensão arterial; DCV: doença cardiovascular; DPOC: doença pulmonar obstrutiva crônica; AVC: acidente vascular cerebral; DAC: doença arterial coronariana; IC: insuficiência cardíaca; IM: injúria miocárdica; nl; normal; tropo: troponina; SDRA: síndrome do desconforto respiratório; UTI: unidade de terapia intensiva.*

Meta-análise recente que incluiu oito estudos da China, com 46.248 pacientes infectados, mostrou que as comorbidades mais prevalentes foram hipertensão (17 ± 7%) e diabetes mellitus (8 ± 6%), seguidas por DCV (5 ± 4%). Wang et al. avaliaram apenas pacientes hospitalizados por COVID-19 e observaram maior prevalência de hipertensão (31,2%), DCV (19,6%) e diabetes (10,1%),^9^ reforçando que os indivíduos com essas comorbidades apresentam forma mais grave da COVID-19, com maior necessidade de internação hospitalar. Esses pacientes evoluíram com mais hipoxemia e maior necessidade de internação em UTI.^9 , 30^ Idade avançada, de modo semelhante, está relacionada à forma grave da doença. A mediana de idade nesses estudos variou de 42 a 64 anos,^11 , 30^ sendo maior em pacientes graves (64 vs 51,5).^29^ A idade também foi mais elevada nos internados em UTI e naqueles com hipoxemia.^9 , 30^

As complicações cardiovasculares também foram frequentes nos pacientes do grupo de risco. Aqueles com DCV cursam com elevação de troponina e maiores taxas de choque e arritmias.^10 - 12^ Guo et al. avaliaram uma coorte com 187 pacientes e observaram que aqueles com injúria miocárdica tinham elevada prevalência de hipertensão (63%vs 28%), diabetes (30,8% vs 8,9%), doença arterial coronária (32,7% vs 3%) e IC (15,4% vs 0%), além de serem mais idosos (mediana 71,4 anos).^10^

Em uma coorte de 191 pacientes, Zhou et al. avaliaram as características daqueles que evoluíram a óbito comparadas às daqueles que receberam alta hospitalar. Nessa coorte, os pacientes que evoluíram a óbito tinham maior prevalência de hipertensão (48%), diabetes (31%) e DCV (24%). A idade avançada foi preditor independente de mortalidade.^12^ A taxa de mortalidade aumenta com o avançar da idade, sendo de 1,3% nos paciente com idade entre 50 e 59 anos, 3,6% naqueles entre 60 e 69 anos, 8% entre 70 e 79 anos e 14,8% em maiores de 80 anos.^31^ Estudos populacionais mostram taxa de mortalidade geral de 6% em hipertensos, 7,3% em diabéticos e 10,5% em pacientes com DCV.^33^

Outro grupo de risco são os pacientes com câncer, que apresentam um maior risco de infecção devido ao comprometimento das defesas do hospedeiro e às sequelas do tratamento antineoplásico. Na China, entre os casos confirmados de COVID-19, a prevalência de câncer variou de 1% a 7%, sendo esse número superior à incidência geral de câncer no país que é de 0,2% (201,7/100.000 pessoas).^2 , 10 , 34^ Esses pacientes evoluíram mais para forma grave da doença em comparação àqueles sem câncer (39% vs 8%).^35^ Entre os indivíduos com câncer submetidos a quimioterapia ou cirurgia recente, 75% desenvolveram doença grave em comparação com 43% daqueles sem tratamento recente.^35^

### Algoritmo de avaliação do sistema cardiovascular

Apesar de não haver recomendações formais sobre a avaliação cardiovascular do paciente com infecção suspeita ou confirmada por SARS-CoV-2, acredita-se que seja benéfica em: a) pacientes que tenham DCV preexistente ou fatores de risco cardiovasculares; b) pacientes que apresentem sinais e sintomas cardiovasculares (dispneia, choque, dor precordial, alteração eletrocardiográfica ou aumento de área cardíaca); c) presença de alteração em biomarcadores como dímero-D, troponina, NT-proBNP e ferritina; e d) pacientes com necessidade de internação. Aqueles com DCV são mais propensos a sofrer injúria miocárdica após infecção por SARS-CoV-2 e apresentam maior risco de morte.^10^ O cardiologista deve fazer parte do time de cuidado do paciente crítico, provendo auxílio na discussão dos casos e no tratamento.

**Figura 3:**
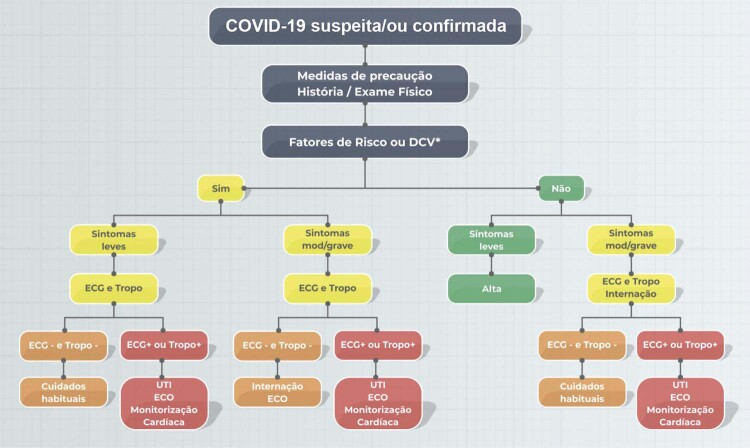
Fluxograma proposto para avaliação cardíaca do paciente com suspeita de COVID-19. *Idade avançada, doença arterial coronária, doença cerebrovascular, hipertensão arterial, diabetes mellitus, cardiomiopatia ou portador de arritmia. COVID-19: doença do Coronavírus 2019; DCV: doença cardiovascular; ECG +: taquicardia supraventricular ou ventricular, alterações de repolarização novas sugestivas de isquemia aguda; ECG -: eletrocardiograma sem alterações agudas; ECO: ecocardiograma; mod: moderados; Tropo +: níveis de troponina maior que o percentil 99 do valor de referência; Tropo -: níveis de troponina abaixo do percentil 99; UTI: Unidade de Terapia Intensiva.

A avaliação cardiológica inicial deve ser realizada por meio de história clínica, exame físico, dosagem de troponina e eletrocardiograma (ECG). A presença de elevação nos níveis de troponina acima do percentil 99 e alterações agudas no ECG auxiliam na identificação dos pacientes de mais alto risco cardiovascular e podem contribuir na decisão de internação hospitalar e condução do caso. A Figura 3 apresenta um fluxograma proposto para avaliação cardíaca nos casos de COVID-19.

O ECG pode identificar arritmias cardíacas malignas, definidas como taquicardia ventricular sustentada induzindo instabilidade hemodinâmica ou fibrilação ventricular. Alterações de repolarização sugestivas de isquemia aguda também foram observadas, especialmente nos pacientes com miocardite.^14 , 18^

O ECG tem papel importante na monitorização do intervalo QTc naqueles em uso de hidroxicloroquina (HCQ) e azitromicina. Ambos os fármacos são relacionados com o prolongamento do intervalo QT. A associação dos fármacos e a presença de alteração hidroeletrolítica em pacientes com COVID-19 requerem a monitorização do intervalo QTc. Naqueles internados, deve-se realizar um ECG de 2 a 3 horas após a segunda dose de HCQ e, depois, diariamente. Se o QTc for >60ms em relação ao ECG basal ou valores absolutos >500ms (ou >530-550ms se QRS >120ms), recomenda-se descontinuar azitromicina ou reduzir a dose de HCQ e realizar ECG diariamente. Se o ECG persistir alterado, deve-se avaliar o risco x benefício de se manter a medicação. Nos pacientes ambulatoriais, com menor risco de complicações do alargamento do QTc, deve-se realizar ECG basal 2 a 3 horas após o início da HCQ e no dia 3 da terapia. Se QTc aumentar > 30-60ms ou se valores absolutos QTc >500ms (ou >530-550ms se QRS >120ms), considerar a descontinuação da terapia.^36^

O ecocardiograma transtorácico deve ser o método de escolha inicial para avaliação da função cardíaca nesses pacientes, devendo ser idealmente realizado na emergência pelo método *point of care* ou dinâmico. O ecocardiograma pode demonstrar comprometimento sistólico e/ou diastólico do ventrículo esquerdo e fornece informações hemodinâmicas que auxiliam no manejo dos pacientes, além de permitir o diagnóstico de alterações pericárdicas. Deve ser considerado em todos os grupos de risco ou naqueles que necessitem de internação hospitalar. Pacientes com disfunção ventricular têm maior probabilidade de precisar de ventilação mecânica e de pior prognóstico.^13^ O acompanhamento do paciente crítico deve ser feito com ecocardiograma diário, avaliação rigorosa dos parâmetros hemodinâmicos e da função biventricular. Além disso, a detecção de disfunção ventricular indicará monitorização hemodinâmica invasiva e guiará o tratamento com suporte inotrópico e/ou circulatório. Nos casos críticos, o ecocardiograma dinâmico deve ser realizado diariamente e a cada mudança hemodinâmica.

A ressonância magnética deve ser considerada em pacientes estáveis e pode auxiliar no diagnóstico diferencial da etiologia da disfunção ventricular, que pode estar relacionada a miocardite ou disfunção sistólica induzida por estresse. O diagnóstico de miocardite segue os critérios clássicos já validados para outras etiologias virais, nas quais podem ser observados edema miocárdico e realce tardio miocárdico de padrão não isquêmico.^37 - 39^

### Manejo do paciente com COVID-19

**Abordagem inicial e suporte intensivo.** O tempo médio de início dos sintomas é de 4 a 5 dias, sendo que 97,5% dos contaminados vão desenvolvê-los em até 11,5 dias da exposição.^32^ A maioria dos pacientes (81%) vai apresentar sintomas leves, os principais sendo febre (88%) e tosse (67,7%). Outros menos frequentes são diarreia, mialgia, cefaleia e coriza. Aproximadamente 20% dos pacientes evoluirão de forma grave, com dispneia, taquipneia, saturação de oxigênio ≤93% e infiltrado pulmonar, e 5% desses apresentarão um quadro crítico, com sinais de choque e falência respiratória.^1 , 40^ Os pacientes assintomáticos ou oligossintomáticos e clinicamente estáveis não necessitam de internação hospitalar, em sua maioria. Aqueles com sintomas graves e evolução desfavorável vão requerer internação hospitalar.

A avaliação inicial deve incluir: ECG, gasometria arterial com lactato, dímero-D, hemograma completo, avaliação de funções renal e hepática, fatores de coagulação, troponina, creatinofosfoquinase, ferritina, DHL, IL-6 e eletrólitos (sódio, magnésio, potássio e cálcio). Deve-se realizar radiografia de tórax e considerar a tomografia computadorizada (TC) de tórax em alguns casos. A TC mostra anormalidades em 85% dos pacientes, observando-se, em 75% deles, envolvimento pulmonar bilateral, comumente caracterizado por áreas de vidro fosco e consolidações subpleurais e periféricas.^41^ Aqueles com indicação de internação deverão ser submetidos a uma ecocardiografia na sala de emergência ou nas primeiras horas da admissão hospitalar.

O curso clínico é variável e potencialmente grave, pois 3,4% dos pacientes evoluem para SDRA,^32^ uma proporção que aumenta nas coortes daqueles internados pela doença (19,6%) e naqueles com injúria miocárdica (58,5%).^9 , 11^ A definição de SDRA é baseada nos critérios de Berlim: início agudo de lesão pulmonar, opacidades pulmonares bilaterais na radiografia de tórax e edema pulmonar. A definição de Berlim da SDRA estratifica a gravidade da lesão pulmonar com base na relação entre a pressão parcial arterial de oxigênio (PaO_2_) e a fração de oxigênio inalado (FiO_2_), medida em uma pressão expiratória final pulmonar (PEEP) ou pressão positiva contínua nas vias aéreas (CPAP) ≥ 5 cmH_2_O. Considera-se SDRA grave quando PaO_2_/FiO_2_ é <100.^42^

A ventilação mecânica é recomendada na presença de hipoxemia apesar da oferta de oxigênio. Estratégias de ventilação mecânica protetora devem ser empregadas, volume corrente de 6 ml/kg, com pressão de platô < 30 cmH_2_O e PEEP ajustada conforme FiO_2_. Os pacientes costumam apresentar boa complacência pulmonar apesar da hipoxemia grave. Naqueles com SDRA e PaO_2_/FiO_2_ ≤ 150, deve-se considerar a posição de pronação. Se houver dissincronia significativa com a ventilação mecânica, bloqueio neuromuscular pode ser realizado.^43^

A monitorização hemodinâmica deve ser cogitada em todos os pacientes internados em UTI e com sinais de choque. A avaliação com monitores de débito minimamente invasivos e a avaliação contínua do débito cardíaco é uma estratégia a se considerar em associação com a ecocardiografia dinâmica e com a análise de marcadores de hipoperfusão tecidual, como parâmetros clínicos e a dosagem do lactato arterial, do deltaPCO_2_ e do excesso de bases. Na presença de choque, a norepinefrina é o fármaco de escolha, sendo recomendada a associação da vasopressina se forem necessárias doses crescentes de noradrenalina para otimização hemodinâmica.^44^ Sugere-se associação da dobutamina nos casos de disfunção cardíaca.^44^ Recomenda-se o início imediato de norepinefrina, mesmo no acesso periférico, evitando-se a hipotensão prolongada, que resulta em elevadas taxas de mortalidade.

A oxigenação por membrana extracorpórea (ECMO) pode ser necessária em pacientes com insuficiência respiratória aguda refratária às medidas iniciais.^45 , 46^ A princípio, a indicação é de uma ECMO venovenosa para recuperação da função pulmonar.^46 , 47^ Quando associada a acometimento cardiovascular importante em pacientes com disfunção ventricular grave e/ou choque cardiogênico, a ECMO venoarterial pode ser considerada.^48^ A ECMO deve ser iniciada antes da instalação de falência múltipla de órgãos.^49^

**Tratamento específico.** Até o momento, o tratamento do paciente crítico tem sido fundamentalmente pautado em medidas de suporte às disfunções orgânicas. Desde o início da pandemia, buscou-se um tratamento antiviral eficaz para a COVID-19. Na China e na Itália, nos casos graves, de uma maneira individualizada a depender da instituição, medicamentos como cloroquina (CQ) ou HCQ, lopinavir/ritonavir, remdesivir e favipiravir têm sido utilizados. Remdesivir e favipiravir são antivirais de amplo espectro, cuja eficácia e segurança no manejo de pacientes com COVID-19 estão sendo avaliadas em ensaios clínicos randomizados.^50^ A combinação lopinavir/ritonavir, utilizada no manejo da infecção pelo HIV, demonstrou, em estudo recente randomizado e controlado, ser ineficaz na infecção pelo SARS-CoV-2.^50^

O difosfato de CQ e o sulfato de HCQ são medicações sabidamente úteis no tratamento da malária e de doenças autoimunes, como artrite reumatoide e lúpus eritematoso sistêmico. Em estudos experimentais, a CQ e a HCQ têm ação contra o SARS-CoV-2, por interferir com a glicosilação da ECA2 e assim reduzir a eficiência da ligação entre a ECA2 das células do hospedeiro e a proteína da superfície do coronavírus. Aqueles fármacos também agem aumentando o pH dos endossomos e lisossomos, através dos quais o processo de fusão do vírus com as células do hospedeiro e a subsequente replicação viral são inibidos. Além disso, a HCQ bloqueia a apresentação de antígeno viral às células T e a transcrição de genes pró-inflamatórios, impedindo a liberação de citocinas. Portanto, em estudos experimentais, a CQ e a HCQ impedem a entrada e a replicação viral além de atenuar a resposta inflamatória. Na China, um estudo mostrou que a CQ estava associada a maior porcentagem de cura clínica e virológica e passou então a ser adotada naquele país no tratamento da COVID-19. Um pequeno estudo relatou que a HCQ com ou sem a azitromicina reduziu a detecção do RNA do SARS-CoV-2 em *swab* respiratório, não tendo sido analisado desfecho clínico.^51 - 53^

Os principais efeitos colaterais dessas medicações são intolerância gastrointestinal (náuseas e vômitos) e, no uso a longo prazo, retinopatia, maculopatia e cardiomiopatia. Efeitos comuns são bloqueio atrioventricular total, bloqueio de ramo, arritmias cardíacas, hipotensão, *torsades de pointes* , inversão de onda T, fibrilação ventricular e taquicardia ventricular, ainda mais frequentes no uso prolongado e na disfunção hepática e renal. Em 10 de março de 2020, uma publicação do *Journal of Critical Care* reuniu a evidência científica a respeito da CQ e da HCQ no tratamento da COVID-19. Foram incluídas recomendações de especialistas, editoriais, estudo *in vitro* e descritos 23 estudos chineses em andamento ou prestes a iniciar o recrutamento.^54^ No dia 21 de março de 2020, o presidente dos Estados Unidos cobrou celeridade do FDA na aprovação do fármaco no tratamento da COVID-19. Entretanto, no momento, o FDA recomenda o uso por compaixão até que tenhamos evidências científicas da eficácia da CQ, da HCQ e da azitromicina no tratamento da COVID-19.

No Brasil, dois estudos foram aprovados pela Comissão Nacional de Ética em Pesquisa (CONEP) no dia 23 de março de 2020: a) estudo de fase IIb para avaliar eficácia e segurança do difosfato de CQ no tratamento de pacientes hospitalizados com SARS-CoV-2: um ensaio clínico, duplo-cego e randomizado – estudo multicêntrico com 440 pacientes proposto pela Diretoria de Ensino e Pesquisa da Fiocruz Amazonas – até o momento incluiu 50 pacientes; e b) avaliação da segurança e da eficácia clínica da HCQ associada à azitromicina em pacientes com pneumonia causada pelo vírus SARS-CoV-2 – estudo multicêntrico com 400 pacientes proposto pela Sociedade Beneficente Israelita Brasileira Albert Einstein – aguardando para iniciar recrutamento.

O Ministério da Saúde do Brasil, a partir do dia 25 de março de 2020, portanto, passa a adotar a medicação como terapia adjuvante no tratamento de formas graves, exclusivamente, sem que outras medidas de suporte sejam preteridas em seu favor, conforme sugestão abaixo. A indicação considera que não existe outro tratamento específico eficaz disponível até o momento e que essa recomendação pode ser modificada a qualquer momento, a depender de novas evidências. Em 31 de março de 2020, em um estudo *preprint* , sem *peer review* , um grupo chinês demonstrou eficácia superior da HCQ em 62 pacientes analisados (com grupo controle) em casos de pneumonia leve.^55^ Esse dado deverá ser confirmado em estudo com maior poder amostral e maior rigor metodológico. Outras medicações em análise são os glicocorticoides, as imunoglobulinas, o interferon e o tocilizumabe.

**Ressuscitação cardiopulmonar.** Quando o paciente com COVID-19 evolui para parada cardiorrespiratória, cuidados especiais devem ser tomados, com atenção no manejo da via aérea por risco maior de contaminação da equipe pela liberação de aerossóis durante as manobras de compressão torácica e ventilação.^56 , 57^ Todos os profissionais de saúde que estão em contato com pacientes devem seguir as orientações locais e nacionais para controle de infecção e uso de equipamentos de proteção individual, que devem estar em locais de fácil acesso.^58 , 59^

Pacientes infectados com SARS-CoV-2, que correm risco de deterioração aguda ou parada cardíaca, devem ser identificados precocemente. Aqueles que foram definidos por quaisquer motivos como ‘não ressuscitação cardiopulmonar’ também devem ser identificados precocemente, e tal definição deve ser baseada nas diretrizes locais vigentes.^58^

A causa mais provável é hipóxia; entretanto, todas as possibilidades devem ser consideradas (hipoglicemia, acidose, trombose coronariana). Os algoritmos já validados na literatura devem ser aplicados conforme a identificação de ritmos chocáveis e não chocáveis.^56 , 57^ A manipulação da via aérea deve ser realizada por profissionais experientes e treinados. As equipes de profissionais que cuidam de pacientes com COVID-19, sejam médicos, enfermeiros ou fisioterapeutas, têm alto risco de contrair a infecção.^60 , 61^ Os procedimentos de geração de aerossóis, como ventilação não invasiva, uso de cânula nasal de alto fluxo, ventilação com bolsa-valva-máscara ou bolsa-tubo endotraqueal, são de risco particularmente alto.^62^

Deve-se evitar a ventilação com bolsa-valva-máscara ou bolsa-tubo endotraqueal, devido ao risco elevado de aerolização e contaminação da equipe e não há evidências de que esse tipo de ventilação seja superior à ventilação mecânica.^56^ Em caso de necessidade de ventilação com bolsa-valva-máscara, deve-se selar corretamente a máscara, sendo necessário o envolvimento de mais de um profissional. Além disso, a utilização de filtros entre a máscara e a bolsa é mandatória. O estabelecimento de via aérea avançada deve ser priorizado nesses pacientes e realizado por indivíduos experientes.^56^ A falha no procedimento de intubação ou impossibilidade requer o auxílio de dispositivos como tubo laríngeo ou máscara laríngea, para que permitam a ventilação mecânica em circuito fechado até que haja possibilidade de acesso definitivo à via aérea, seja por intubação traqueal ou por cricotireoidostomia.^57^

**Figura 4:**
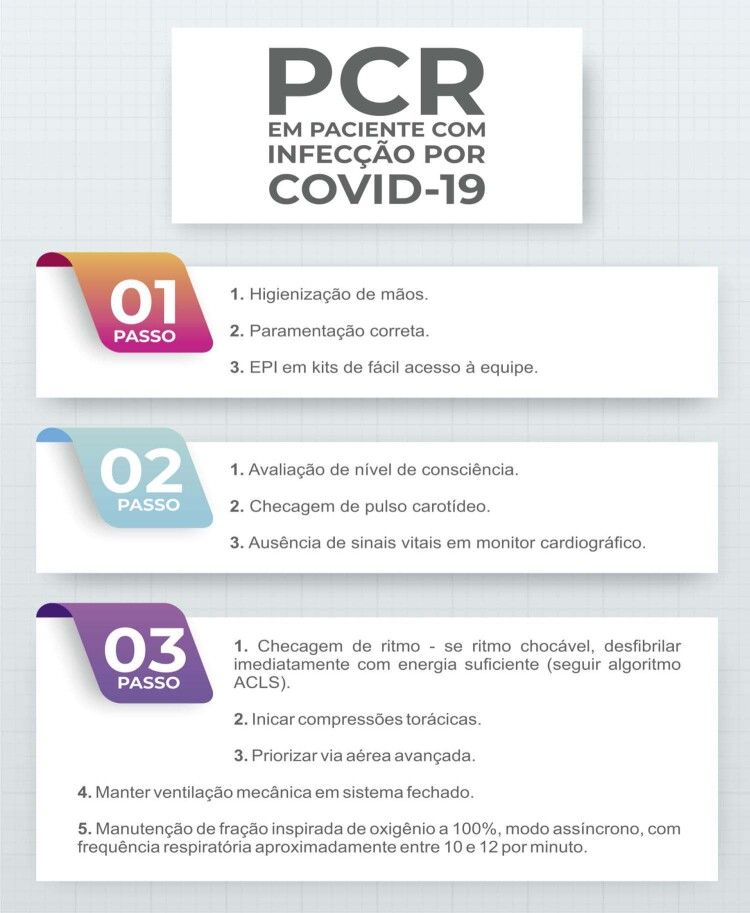
Ressuscitação cardiopulmonar em paciente com COVID-19. PCR: parada cardiorrespiratória; COVID-19: doença do Coronavírus 2019; EPI: equipamento de proteção individual; ACLS: Advanced Cardiovascular Life Support.

No caso de parada cardiorrespiratória em pacientes sob ventilação mecânica, para que não haja contaminação por aerossóis pelas manobras de reanimação cardiopulmonar e ventilação, deve-se manter o paciente conectado ao ventilador mecânico em circuito fechado, com manutenção de FiO_2_ de 100%, modo assíncrono, com frequência respiratória de 10 a 12 incursões por minuto ( Figura 4 ).^56^

### Prevenção e manejo da trombose

A literatura sugere que a exacerbada resposta inflamatória sistêmica presente na COVID-19 possa causar disfunção endotelial e aumento da atividade pró-coagulante, o que associado à menor oferta de oxigênio pode contribuir para a formação de trombo sobre uma placa coronariana rompida ou para a instabilidade de placa coronariana, tornando-a vulnerável.^10 , 11 , 63^ É importante o diagnóstico diferencial da coronariopatia obstrutiva com infarto do miocárdio tipo II.^64^ O paciente com COVID-19 pode apresentar uma síndrome coronariana aguda por desequilíbrio entre oferta e demanda de oxigênio ao miocárdio, sendo diagnosticado com infarto tipo II. Deve-se analisar individualmente os casos, porque boa parte deverá ser manejada com uma estratégia conservadora, pois cerca de 7% dos pacientes com COVID-19 e síndrome coronariana aguda podem ter infarto do miocárdio tipo II ou miocardite.^64^

A definição da abordagem da síndrome coronariana aguda no paciente com COVID-19 deve levar em consideração os recursos locais disponíveis, como serviços de hemodinâmica estruturados, leito de unidade coronariana e/ou UTI e adequação do ambiente às medidas de proteção ao SARS-CoV-2.^64^ Um relatório chinês sugeriu que fosse considerada a trombólise como terapia de primeira escolha nos pacientes com COVID-19. Essa recomendação é controversa, especialmente em locais em que a angioplastia primária possa ser realizada, respeitando todas as regras de segurança exigidas para proteção dos profissionais de saúde e do ambiente hospitalar (uso de equipamentos de proteção individual, sala com pressão negativa, limpeza adequada).^64^

O tratamento das complicações cardiovasculares deve ser baseado no uso ideal e criterioso das terapias indicadas nas diretrizes. A abordagem terapêutica com IECA, BRA, betabloqueadores, agentes antiplaquetários e estatinas deve seguir as indicações das diretrizes, respeitando as contraindicações presentes, referentes a estabilidade hemodinâmica e presença de outras disfunções orgânicas.^21^

Pacientes com COVID-19 são de elevado risco para tromboembolismo venoso, devido à prolongada redução de mobilidade e parâmetros anormais da coagulação.^4^ Recomenda-se o uso de estratégias de prevenção não farmacológica para todos aqueles internados por COVID-19. Estratégias farmacológicas devem ser consideradas, como o uso de heparina não fracionada ou de heparina de baixo peso molecular, atentando-se para suas contraindicações e a depuração de creatinina do paciente. A suspeita de tromboembolismo venoso deve ser feita de acordo com critérios clínicos, ou em situações como manutenção de altos níveis de dímero-D, em hipoxemia refratária ou em se detectando sinais de hipertensão pulmonar e de disfunção de ventrículo direito ao ecocardiograma.

### Telemedicina e cardiologia

Em virtude do crescimento exponencial da disseminação do vírus, foi determinado que o distanciamento social é um fator-chave na diminuição da velocidade de transmissão por diminuir o contato pessoa a pessoa. Dessa maneira, torna-se indispensável o uso de tecnologia da informação como uma resposta de emergência às questões ambientais ou riscos biológicos presentes. A telessaúde permite a triagem remota, auxilia no diagnóstico de doenças e garante o acesso aos cuidados de rotina durante um surto de doença infecciosa.^65^

Em 2019, o Conselho Federal de Medicina publicou um decreto que definiu a telemedicina como o exercício da medicina mediado por tecnologias para fins de assistência, educação, pesquisa, prevenção de doenças e lesões e promoção de saúde, regulamentando essa prática. A Sociedade Brasileira de Cardiologia publicou uma diretriz em telemedicina aplicada à cardiologia, também designada telecardiologia. A telecardiologia por meio de suas múltiplas ações na promoção da saúde, prevenção de doenças, diagnóstico, tratamento e reabilitação, com impacto na melhora da qualidade de vida, pode ser considerada uma importante aliada do sistema de saúde, seja ele público, suplementar ou privado, para promover atenção à saúde integral e com qualidade. A implementação da telecardiologia é importante tanto na atenção primária de saúde quanto na atenção especializada.^66^ Na cardiologia, a telemedicina pode ser útil no controle dos fatores de risco como pressão arterial, diabetes mellitus, melhora do perfil lipídico, redução de peso e aumento do sucesso de programas de cessação do tabagismo.^66^

No momento atual de controle da pandemia, a telemedicina torna-se uma ferramenta útil, especialmente para os pacientes de alto risco, diminuindo a exposição a contaminações pelo SARS-CoV-2 e auxiliando no controle das comorbidades. Em 19 de março de 2020, frente a essa pandemia, o Conselho Federal de Medicina, de acordo com o Ministério de Saúde, reconhece a possibilidade e a eticidade da telemedicina, nos termos da teleorientação, teleconsulta e telemonitoramento.^67^

### Recomendações gerais

- Intensificar os cuidados e as medidas de prevenção contra a infecção pelo novo coronavírus na população de pacientes portadores de DCV.
- Os pacientes cardiopatas devem ser conduzidos de acordo com as diretrizes vigentes, assegurando-se o melhor tratamento disponível para as enfermidades crônicas.
- Considera-se fundamental que os pacientes portadores de DCV mantenham rigorosa aderência a dieta adequada, sono regular e atividade física, evitando exposição ao tabagismo e ao etilismo.
- A atualização das vacinas é importante, incluindo pneumocócica, devido ao risco aumentado de infecção bacteriana secundária pelo SARS-CoV-2, além da vacina contra influenza, que é indicada para os pacientes com DCV.
- Recomenda-se o adiamento das consultas ambulatoriais e dos exames e procedimentos eletivos, se, ao julgamento clínico, esses procedimentos não forem essenciais naquele momento e a sua não realização não aumentar o risco de eventos ou prejudicar a condução clínica das DCV de base do paciente. A telemedicina deve ser utilizada como opção para seguimento dos pacientes.
- Recomenda-se redução da quantidade de profissionais de saúde que participam das visitas dos pacientes e o desenvolvimento de discussões *online* .

## Conclusões

A COVID-19 é potencialmente grave e apresenta elevado índice de disseminação. Os dados atuais disponíveis são de estudos predominantemente retrospectivos, que devem ser interpretados com cautela. Entretanto, as evidências atuais já mostram a necessidade de atenção especial aos pacientes do grupo de risco e a importância de um manejo adequado das complicações cardiovasculares, com rápida identificação e implementação de tratamento adequado.
