# Supplementary material for: O Coração e a COVID-19: O que o Cardiologista Precisa Saber
Source: Arq Bras Cardiol. 2020 May 22;114(5):805–16. [Article in Portuguese] doi: 10.36660/abc.20200279 (PMC8386992; doi:10.36660/abc.20200279)
Supplement: Translation

# Translation: The Heart and COVID-19: What Cardiologists Need to Know

**Authors:** Isabela Bispo Santos da Silva Costa, Cristina Salvadori Bittar, Stephanie Itala Rizk, Antônio Everaldo de Araújo, Karen Alcântara Queiroz Santos, Theuran Inahja Vicente Machado, Fernanda Thereza de Almeida Andrade, Thalita Barbosa González, Andrea Nataly Galarza Arévalo, Juliano Pinheiro de Almeida, Fernando Bacal, Gláucia Maria Moraes de Oliveira, Marcus Vinícius Guimarães de Lacerda, Silvio Henrique Barberato, Antonio Carlos Palandri Chagas, Carlos Eduardo Rochitte, José Antonio Franchini Ramires, Roberto Kalil, Ludhmila Abrahão Hajjar

**Affiliations:** 1 Brazil Universidade de São Paulo - Instituto do Câncer do Estado de São Paulo, São Paulo, SP - Brazil; 2 Brazil Universidade de São Paulo Instituto do Coração, São Paulo, SP - Brazil; 3 Brazil Universidade Federal do Rio de Janeiro – Cardiologia, Rio de Janeiro, RJ - Brazil; 4 Brazil Fundação de Medicina Tropical Doutor Heitor Vieira Dourado, Manaus, AM - Brazil; 5 Brazil CardioEco Centro de Diagnóstico Cardiovascular, Curitiba, PR - Brazil; 6 Brazil Quanta Diagnóstico – Ecocardiografia, Curitiba, PR - Brazil; 7 Brazil Faculdade de Medicina da Fundação do ABC, Santo André, SP - Brazil; 8 Brazil Hospital do Coração, São Paulo, SP – Brazil

**Keywords:** Coronavirus, COVID-19, Cardiovascular Diseases/complications, Heart/physiopathology, Pandemics, Respiratory Distress Syndrome, Adult, Risk Factors, Patient Care

## Abstract

In face of the pandemic of the novel coronavirus disease 2019 (COVID-19), the management of patients with cardiovascular risk factors and/or disease is challenging. The cardiovascular complications evidenced in patients with COVID-19 derive from several mechanisms, ranging from direct viral injury to complications secondary to the inflammatory and thrombotic responses to the infection. The proper care of patients with COVID-19 requires special attention to the cardiovascular system aimed at better outcomes.

## Introduction

Currently the world faces the pandemic of the novel coronavirus disease 2019 (COVID-19), which emerged in December 2019 in the city of Wuhan, province of Hubei, in China.^1 , 2^ The initial cases were described as pneumonia that rapidly progressed to acute respiratory distress syndrome (ARDS).

This novel virus, named severe acute respiratory syndrome coronavirus 2 (SARS-CoV-2), is the seventh coronavirus identified so far and differs from the other coronaviruses that cause common cold and mild pneumonia (229E, OC43, NL63 and HKU1). The SARS-CoV-2 is similar to the severe acute respiratory syndrome coronavirus (SARS-CoV) and the Middle East respiratory syndrome coronavirus (MERS-CoV), responsible for the infections occurring in China in 2002-2003 and in the Middle East in 2012, respectively.^1 , 2^ Despite the phylogenetic similarities between SARS-CoV-2 and the zoonotic coronaviruses that caused SARS and MERS, the SARS-CoV-2 spread is much higher, contributing to an infection dissemination ten times faster than that of the SARS-CoV.^2 - 4^ The basic reproduction number (R0) of COVID-19 is 2.78, meaning that, on average, each individual infected can transmit the disease to three others.^5^ A study recently published in *Science* has stated, by use of a mathematical model, that 85% of the COVID-19 transmissions occur from asymptomatic individuals.^6^

Because of its fast dissemination, COVID-19 was declared a pandemic by the World Health Organization (WHO) on March 11, 2020.^4^ At the current time, COVID-19 affects more than 181 countries and the number of cases keeps increasing exponentially. Up to April 2, 2020, 1,015,403 cases and 53,030 deaths had been registered around the world, yielding a lethality of 5.2%. Up to that same date, 8,044 confirmed cases and 324 deaths had been registered in Brazil, with a mortality of 4%. Initial Brazilian data have shown 90% of the deaths occurring among individuals aged over 60 years and 84% of the patients with at least one comorbidity, 51% with cardiovascular disease (CVD) and 37.7% with diabetes.^7^

The analysis of 44,672 confirmed cases of COVID-19 in Wuhan has evidenced an overall case-fatality rate of 2.3%; however, among those with preexisting comorbidities, the case-fatality rate was higher: 10.5% for CVD, 7.3% for diabetes and 6% for hypertension.^8^ In addition, cardiovascular complications due to COVID-19, such as myocardial injury (20% of the cases), arrhythmias (16%), myocarditis (10%), heart failure (HF) and shock (up to 5% of the cases), have been reported.^9 - 11^

This review was aimed at aiding healthcare professionals (clinicians, emergencists, cardiologists and intensivists) involved in the care of patients with COVID-19, proposing an algorithm of cardiovascular assessment for the early detection of complications, in addition to recommending protocols to treat cardiovascular complications in those patients.

### Complications of COVID-19 on the cardiovascular system

Recent data of the COVID-19 pandemic have shown that the virus can affect the cardiovascular system with several manifestations, such as myocardial injury, HF, Takotsubo syndrome (TS), arrhythmias, myocarditis and shock.^4 , 11 - 14^ The damage due to COVID-19 to the cardiovascular system is probably multifactorial and can result from an imbalance between high metabolic demand and low cardiac reserve, systemic inflammation and thrombogenesis, in addition to direct cardiac damage from the virus.^13^ This damage to the cardiovascular system occurs mainly in patients with cardiovascular risk factors (advanced age, hypertension and diabetes) or preexisting CVD.^10 , 11^Figure 1 summarizes the inflammatory response to the viral infection, which leads to damage to the cardiovascular system and lungs, with elevation in the levels of d-dimer, procalcitonin, C-reactive protein, ferritin, troponin and NT-proBNP, culminating in cardiovascular complications and death.

**Figure 1:** Coronavirus and the heart. Patients with risk factors and/or cardiovascular disease are prone to develop severe forms of COVID-19 and its complications. Pulmonary impairment manifests initially as an influenza syndrome (cough and fever), progressing to pneumonia (dyspnea, hypoxemia, tachypnea) and, in some cases, to ARDS. Host response to the virus leads to systemic inflammation findings, with elevation of markers of inflammation (CRP, procalcitonin, d-dimer, IL-6, ferritin, LDH) and of myocardial injury / cardiac dysfunction (troponin/NT-proBNP), which predisposes to acute heart failure, myocarditis, thrombosis and arrhythmias. Cardiovascular complications hinder the host response to the virus, leading to shock, failure of multiple organs and death. CAD: coronary artery disease; LDH: lactate dehydrogenase; LVEF: left ventricular ejection fraction; CRP: C-reactive protein; IL-6: interleukin-6; ARDS: acute respiratory distress syndrome.

The systemic inflammatory response to SARS-CoV-2 is accompanied by higher concentrations of cytokines related to injury to the cardiovascular system.^15^ The increase in troponin levels is accompanied by an elevation in other inflammatory markers, such as d-dimer, ferritin, interleukin 6 (IL-6), lactate dehydrogenase (LDH), C-reactive protein, procalcitonin and leukocyte count.^1 , 11^ Zhou et al. have shown higher levels of d-dimer, IL-6, ferritin and LDH, as well as lymphopenia, in patients who died, suggesting that those inflammatory markers might have prognostic implications. A d-dimer level at admission greater than 1µg/mL was an independent predictor of mortality in that population.^12^ In addition to elevated inflammatory markers, patients with COVID-19 show increased BNP or NT-proBNP levels, markers of myocardial dysfunction. Patients with myocardial injury showed higher NT-proBNP levels, with positive linear correlation.^10 , 11^ This finding reinforces that those with myocardial injury are prone to cardiac function impairment.^10^

A meta-analysis with four studies, including 341 COVID-19 patients, has reported significantly higher troponin I levels in patients with severe disease as compared to those with non-severe disease.^16^ Patients with myocardial injury more often required admission to the intensive care unit (ICU) (22.2% vs. 2.0%), had a higher incidence of HF (52% vs 12%) and a higher death rate (59% vs. 1%).^1 , 9^ Shi et al., assessing 416 hospitalized patients with COVID-19, have reported that myocardial injury, defined as troponin levels above the 99th-percentile upper reference limit, is a frequent complication (19.7%) in those patients, being associated with increased mortality and ARDS.^11^ On multivariate analysis, myocardial injury and ARDS were independent predictors of mortality (HR 4.26 and 7.89, respectively).^11^ In a recent study, Guo et al. have reported elevated troponin levels in 27.8% of 187 patients with COVID-19. Among patients without CVD and with normal troponin levels, mortality was 7.6%; among patients with CVD and normal troponin levels, mortality was 13.3%; among patients without CVD and with elevated troponin levels, mortality was 37.5%; and among patients with CVD and elevated troponin levels, mortality was 69.4%. There was a strong correlation between high troponin levels and increased C-reactive protein and NT-proBNP levels. Patients with increased troponin levels had a higher incidence of ventricular arrhythmias and higher need for mechanical ventilation.^10^

Cardiovascular complications, such as HF, myocarditis, acute myocardial infarction, shock and arrhythmias, are also frequent in patients with myocardial injury. In a cohort with 150 patients, 7% of them developed irreversible myocardial damage and HF, associated with significant elevations in troponin levels.^17^ Malignant arrhythmias (ventricular tachycardia with degeneration to ventricular fibrillation or hemodynamic instability) have been most frequently observed in individuals with troponin elevation (11.5% vs 5.2%).^10^ Patients with severe COVID-19 can rapidly develop important cardiovascular impairment, shock and failure of multiple organs. In two Chinese cohorts of hospitalized patients with COVID-19, up to 20% developed the severe form of disease with shock.^9 , 12^

Myocarditis can be related to acute HF in patients with COVID-19. Cases of COVID-19-related myocarditis have been described, with fulminant myocarditis, rapid progression and significant ventricular dysfunction, associated with diffuse myocardial edema. Those patients had electrocardiographic changes and troponin elevation.^14 , 18 , 19^ Although TS has not been directly linked to COVID-19, some cases of ventricular dysfunction in COVID-19 patients might be attributed to that syndrome, which is a frequent complication in individuals with exacerbated systemic inflammatory response, in whom the stress and severity of the viral infection trigger the TS.^20^

### Interaction of SARS-CoV-2 with angiotensin-converting enzyme-2

Some studies have suggested that the damage to the cardiovascular system secondary to SARS-CoV-2 can be linked to the angiotensin-converting enzyme-2 (ACE2),^13 , 15^ which is related to the immune system and present in high concentration in the lungs and heart. The ACE2 down-regulates the angiotensin-renin system by inactivating angiotensin-2, and ACE2 might have a protective role against the development of respiratory failure and its progression. SARS-CoV-2 has four main structural proteins: spike (S), nucleocapsid (N), membrane (M), and envelope (E) proteins. The coronavirus spike protein binds to the ACE2 receptor and the virus enters the host cell ( Figure 2 ), where ACE2 inactivation occurs, favoring pulmonary damage. Because of the high ACE2 concentrations in the heart, potentially severe damage to the cardiovascular system can occur.^13 , 21^

**Figure 2:** The SARS-CoV-2, via its surface spike protein, binds to the human ACE2 receptor after spike protein activation by TMPRSS2. SARS-CoV: severe acute respiratory syndrome coronavirus; SARS-COV-2: severe acute respiratory syndrome coronavirus 2; COVID-19: coronavirus disease 2019; ACE2: angiotensin-converting enzyme-2; TMPRSS2: transmembrane protease serine-2.

Patients with preexisting CVD apparently have increased serum levels of ACE2, which might contribute to the more severe manifestations in that population.^22 - 24^ Similarly, individuals with hypertension would have a higher ACE2 expression secondary to the use of angiotensin-converting enzyme inhibitors (ACEI) or angiotensin II receptor blockers (ARB), which would potentially increase the susceptibility to SARS-CoV-2 infection.^4^ However, current studies on humans have some limitations: a) assessment of a small number of individuals using those drugs, and b) the advanced age of a large part of the patients assessed, which is an important confounder, because advanced age increases the susceptibility to infection and is the major factor of poor prognosis.^25^

It is worth noting that, despite substantial structural homology between ACE2 and ACE, their enzyme active sites are distinct, and, as a result, ACE inhibitors in clinical use do not directly affect ACE2 activity. In addition, that enzyme plays a well-known role in the recovery of ventricular function of patients with myocardial injury, because it inhibits angiotensin II activity.^26^ On the other hand, angiotensin II has been suggested to account for the cardiac injury by the coronavirus, and the administration of recombinant ACE2 would normalize angiotensin II levels. Studies with recombinant ACE2 and losartan are being conducted.^25^

The current recommendation is that ACEI and ARB should be continued in patients on regular use of those drugs, because of the clear benefit of blood pressure control and mortality decrease in those with HF, as evidenced in randomized studies.^27 , 28^ In the severe forms of COVID-19, hemodynamic stability and renal function should be assessed individually before deciding on the continuation or withdrawal of the drugs.

### Cardiovascular disease as a risk for the severe form of COVID-19

Patients with cardiovascular risk factors (advanced age, hypertension and diabetes), as well as those with CVD (coronary artery disease, cardiomyopathies and cerebrovascular disease), have susceptibility to the severe form of COVID-19 and cardiovascular complications, being classified as a risk group. Approximately 80% of the patients with the severe form of COVID-19 have a comorbidity.^29^Table 1 summarizes the major studies that characterize the clinical comorbidities of patients with COVID-19.^9 - 12 , 17 , 29 - 32^

**Table 1:** Summary of the clinical characteristics of the major studies on COVID-19

Author	N	Type	Age (years)	Comorbidities	Major findings
Huang et al. 2020^17^	41	Prospective	49 (41-58)	- DM: 8 (20%) - AH: 6 (15%) - CVD: 6 (15%) - COPD: 1 (2%) - Cancer: 1 (2%)	- 13 (32%) ICU admissions - 5 (12%) MI, and 4 (31%) to the ICU - 3 (7%) shock and 12 (29%) ARDS - Mortality: 6 (15%)
Wang et al. 2020^30^	69	Retrospective	42 (35-62)	- AH: 9 (13%) - CVD: 8 (12%) - DM: 7 (10%) - COPD: 4 (6%) - Cancer: 4 (6%)	- Hospitalizations: 44 (65.7%) - Mortality: 5 (7.5%) - Patients with DM, AH and CVD more often had hypoxemia (SatO_2_< 90%) - MI not assessed
Chen et al. 2020^31^	99	Retrospective	55 (21-82)	- CVD: 40 (40%) - DM: 12 (12%) - Cancer: 1 (1%)	- 57 (58%) hospitalizations, 17 (17%) ARDS, 4 (4%) shock - Mortality: 11 (11%) - Of the deceased, 63% were > 60 years and 33% had AH
Wang et al. 2020^9^	138	Retrospective	56 (42-68)	- AH: 43 (31.2%) - CVD: 20 (14.5%) - DM: 14 (10.1%) - Cancer: 10 (7.2%) - Stroke: 7 (5.1%)	- 36 (26%) ICU admissions, high prevalence of risk factors - 12 (8.7%) shock, 23 (16.7%) arrhythmias, 27 (19.6%) ARDS, and 10 (7.2%) MI - Mortality: 6 (4.3%)
Zhang et al. 2020^29^	140	Retrospective	57 (20–83)	- AH: 42 (30%) - DM: 17 (12.1%) - CAD: 7 (5%) - Arrhythmias: 5 (3.6%)	- Comparing severe x non-severe groups: median age 64 vs 51.5, p < 0.001 comorbidities 79.3% vs 53.7%, p = 0.002 d-dimer 0.4 vs 0.2, p<0.001
Guo et al. 2020^10^	187	Retrospective	58.5 (±14.7)	- AH: 61 (32.6%) - CAD: 21 (11.2%) - HF: 8 (4.3%) - DM: 28 (15%) - COPD: 4 (2.1%) - Cancer: 13 (7%)	- 52 (27.8%) MI - Comparing normal tropo x high tropo: AH: 27% vs 63.5%, p 0.001 CAD: 3% vs 32.7%, p <0.001 HF: 0% vs 15.4%, p <0.001 - 43 deaths, 31 (59.6%) in the MI group - Mortality: 13.3% CVD without MI, and 69.4% CVD with MI
Zhou et al 2020^12^	191	Retrospective	56 (46-67)	- AH: 58 (30%) - DM: 36 (19%) - CAD: 15 (8%) - COPD: 6 (3%) - Cancer: 2(1%)	- MI: 24/145 (17%), greater in patients who died (22.2 [5.6-83.1] vs 3.0 [1.1-5.5], p <0.001) - HF 44 (23%), shock 38 (20%), ARDS 59 (31%) - 54 (28%) deaths, 67% with comorbidities
Shi et al. 2020^11^	416	Prospective	64 (21-95)	- AH: 127 (30.5%) - DM: 60 (14.4%) - CAD: 44 (10.6%) - Stroke: 22 (5.3%) - HF: 17 (4.1%) - Cancer: 9 (2.2%)	- 82 (19.7%) MI - High prevalence of AH, DM, CAD and HF in patients with MI - MI was related to higher mortality: (42 of 82 [51.2%] vs 15 of 334 [4.5%]; p < .001) - MI was associated with ARDS: (48 of 82 [58.5%] vs 49 of 334 [14.7%]; p < .001)
Guan et al. 2020^32^	1099	Retrospective	47 (35-58)	- COPD: 12 (1.1%) - DM: 81 (7.4%) - AH: 165 (15%) - CAD: 27 (2.5%) - Stroke: 15 (1.4%) - Câncer: 10 (0.9%)	- Severely-ill patients: AH 41 (23.7%) - High CK-MB 90/657 (13.7%) - 12 (1.1%) shock, 37 (3.4%) ARDS, 1029 (93.6%) hospitalizations, 55 (5%) ICU admissions - Mortality: 15 (1.4%)

*DM, diabetes mellitus; AH, arterial hypertension; CVD, cardiovascular disease; COPD, chronic obstructive pulmonary disease; CAD, coronary artery disease; HF, heart failure; MI, myocardial injury; tropo, troponin; ARDS, acute respiratory distress syndrome; ICU, intensive care unit.*

A recent meta-analysis including eight studies from China, with 46,248 infected individuals, has shown that the most prevalent comorbidities were hypertension (17 ± 7%), diabetes mellitus (8 ± 6%) and CVD (5 ± 4%). Wang et al., assessing only hospitalized patients with COVID-19, have reported a higher prevalence of hypertension (31.2%), CVD (19.6%) and diabetes (10.1%),^9^ emphasizing that individuals with those comorbidities have the most severe form of COVID-19, usually requiring hospitalization. These patients more often had hypoxemia and need for ICU admission.^9 , 30^ Likewise, advanced age is related to the severe form of disease. In those studies, the median age has ranged from 42 to 64 years,^11 , 30^ being higher in severely ill patients (64 vs 51.5).^29^ In addition, patients admitted to ICU and those with hypoxemia were older.^9 , 30^

Cardiovascular complications were also frequent among patients from the risk group. Those with CVD had troponin elevation and higher rates of shock and arrhythmias.^10 - 12^ Guo et al., assessing a cohort with 187 patients, have observed that those with myocardial injury had a high prevalence of hypertension (63%vs 28%), diabetes (30.8% vs 8.9%), coronary artery disease (32.7% vs 3%) and HF (15.4% vs 0%), and were older (median age, 71.4 years).^10^

In a cohort of 191 patients, Zhou et al. have assessed the characteristics of the deceased ones as compared to those of the discharged ones. In that cohort, the deceased patients had a higher prevalence of hypertension (48%), diabetes (31%) and CVD (24%). Advanced age was an independent predictor of mortality.^12^ Mortality rate increases with increasing age as follows: 1.3% in patients aged 50-59 years; 3.6% in patients aged 60-69 years; 8% in patients aged 70-79 years; and 14.8% in patients aged 80 years and older.^31^ Population studies have reported an overall mortality rate of 6% in patients with hypertension, 7.3% in patients with diabetes and 10.5% in patients with CVD.^33^

Patients with cancer have a higher risk for COVID-19 because of their impaired defense and their sequelae from the antineoplastic treatment. In China, among the confirmed cases of COVID-19, the prevalence of cancer has ranged from 1% to 7%, which is higher than the overall incidence of cancer in that country (0.2% - 201.7/100,000 individuals).^2 , 10 , 34^ Patients with cancer more often developed the severe form of COVID-19 as compared to those without cancer (39% vs 8%).^35^ Of the patients with cancer submitted to recent chemotherapy or surgery, 75% developed severe disease as compared to 43% of those with no recent treatment.^35^

### Algorithm of cardiovascular assessment

Although not formally, cardiovascular assessment of patients with suspected or confirmed SARS-CoV-2 infection is recommended in the following situations: a) preexisting CVD or cardiovascular risk factors; b) cardiovascular signs and symptoms (dyspnea, shock, chest pain, electrocardiographic alterations or increased cardiac area); c) alterations on biomarkers, such as d-dimer, troponin, NT-proBNP and ferritin; and d) need for hospitalization. Those with CVD are prone to experience myocardial injury after SARS-CoV-2 infection, in addition to being at a higher risk of death.^10^ Cardiologists should be part of the team caring for critical patients, aiding in clinical discussions and treatment.

The initial cardiovascular assessment should comprise clinical history, physical examination, troponin levels, and electrocardiogram (ECG). Troponin levels above the 99th-percentile upper reference limit and acute alterations on ECG support the identification of patients at higher cardiovascular risk and might contribute to the decision making on hospital admission and case management. Figure 3 shows the flowchart for cardiovascular assessment in COVID-19 cases.

**Figure 3:** Flowchart for cardiac assessment of patients with suspected COVID-19. *Advanced age, coronary artery disease, cerebrovascular disease, arterial hypertension, diabetes mellitus, cardiomyopathy or arrhythmia. COVID-19: Coronavirus disease 2019; CVD: cardiovascular disease; ECG +: supraventricular or ventricular tachycardia, new repolarization changes suggestive of acute ischemia; ECG -: electrocardiogram without acute changes; ECHO: echocardiogram; mod: moderate; Tropo +: troponin levels above the 99th-percentile upper reference limit; Tropo -: troponin levels below the 99th-percentile; ICU: intensive care unit.

The ECG can identify malignant cardiac arrhythmias, defined as sustained ventricular tachycardia inducing hemodynamic instability or ventricular fibrillation. Alterations in repolarization suggesting acute ischemia have been reported, mainly in patients with myocarditis.^14 , 18^

The ECG plays an important role in the QTc interval monitoring of patients on hydroxychloroquine (HCQ) and azithromycin. Both drugs have been linked to QT interval prolongation. The combination of both drugs and the presence of fluid and electrolyte imbalance in patients with COVID-19 require QTc interval monitoring. In-patients should undergo an ECG 2-3 hours after the second dose of HCQ and daily thereafter. If QTc increases by >60ms or absolute QTc >500ms (or >530-550ms if QRS >120ms), azithromycin should be discontinued or the HCQ dose, reduced, and ECG performed daily. If the ECG changes remain, the risk/benefit of maintaining the medication should be reevaluated. For outpatients, who may be less at risk for complications from QT interval prolongation, baseline ECG should be acquired 2-3 hours after initiating HCQ and on day 3 of therapy. If QTc increases by >30-60ms or absolute QTc >500ms (or >530-550ms if QRS >120ms), consider discontinuing therapy.^36^

Transthoracic echocardiogram should be the initial choice for assessing cardiac function in those patients, and ideally performed at the emergency department by use of the point-of-care or dynamic method. Transthoracic echocardiogram can show systolic and/or diastolic left ventricular impairment and provides hemodynamic information to support the management of patients, in addition to enabling the diagnosis of pericardial changes. It should be considered for all risk groups or those requiring hospitalization. Patients with ventricular dysfunction are more likely to need mechanical ventilation and be of worse prognosis.^13^ Critical patients should be followed up with daily echocardiogram, as well as strict assessment of hemodynamic parameters and biventricular function. In addition, the detection of ventricular dysfunction is an indication for invasive hemodynamic monitoring and will guide the treatment with inotropic and/or circulatory support. In critical cases, dynamic echocardiogram should be acquired daily and at every hemodynamic change.

Magnetic resonance imaging should be considered in stable patients and can support the differential diagnosis of ventricular dysfunction etiology, which might be related to myocarditis or stress-induced systolic dysfunction. The diagnosis of myocarditis follows the classic criteria already validated for other viral etiologies, in which myocardial edema and non-ischemic myocardial late enhancement can be observed.^37 - 39^

### Management of the patient with COVID-19

**Initial approach and intensive support.** The mean time of symptom onset is 4-5 days, and 97.5% of contaminated individuals will have symptoms in up to 11.5 days from exposure.^32^ Most patients (81%) have mild symptoms, the most common being fever (88%) and cough (67.7%). Other less frequent are diarrhea, myalgia, headache and runny nose. Approximately 20% of the patients with COVID-19 will have the severe form, with dyspnea, tachypnea, oxygen saturation ≤93%, and pulmonary infiltrate, while 5% will have the critical form of COVID-19, with signs of shock and respiratory failure.^1 , 40^ Most of asymptomatic or olygosymptomatic clinically stable patients require no hospitalization, which is mandatory for those with severe symptoms and unfavorable evolution.

The initial assessment of patients with COVID-19 should include: ECG, arterial blood gas analysis with lactate level, d-dimer, complete blood count, kidney and liver function tests, clotting factors, troponin, creatine phosphokinase, ferritin, LDH, IL-6 and electrolytes (sodium, magnesium, potassium, and calcium). Chest radiography should be performed and chest computed tomography (CT) considered in some cases. Computed tomography evidences abnormalities in 85% of the patients, and 75% of them show bilateral pulmonary involvement, commonly characterized as ground-glass opacifications and subpleural and peripheral consolidations.^41^ Those with indication for hospitalization should undergo echocardiography in the emergency department or within the first hours from hospital admission.

The clinical course of COVID-19 is variable and potentially severe, because 3.4% of the patients progress to ARDS,^32^ a proportion that increases in the cohorts of those hospitalized with the disease (19.6%) and among those with myocardial injury (58.5%).^9 , 11^ Acute respiratory distress syndrome is defined based on the Berlin criteria: acute onset of pulmonary damage, bilateral pulmonary opacities on chest radiography, and pulmonary edema. The ARDS Berlin Definition stratifies the severity of pulmonary damage based on the relation between partial pressure of arterial oxygen (PaO_2_) and fraction of inspired oxygen (FiO_2_), acquired in a positive-end expiratory pressure (PEEP) or continuous positive airway pressure (CPAP) ≥ 5 cm H_2_O. The ARDS is considered severe when PaO_2_/FiO_2_is <100.^42^

Mechanical ventilation is recommended in the presence of hypoxemia despite oxygen supply. Protective mechanical ventilation strategies should be used, with tidal volume of 6 mL/kg, plateau pressure < 30 cm H_2_O, and PEEP adjusted according to FiO_2_. Patients usually have good pulmonary compliance despite severe hypoxemia. For patients with ARDS and PaO_2_/FiO_2_≤ 150, prone position should be considered, and, in case of significant patient-ventilator dyssynchrony, neuromuscular block can be performed.^43^

Hemodynamic monitoring should be cogitated in all ICU patients with signs of shock. Minimally invasive hemodynamic monitoring and continuous cardiac output monitoring should be considered in association with dynamic echocardiography and analysis of tissue hypoperfusion markers, such as clinical parameters, arterial lactate levels, deltaPCO_2_, and base excess. In the presence of shock, norepinephrine is the drug of choice, and the addition of vasopressin is recommended if increasing doses of noradrenaline are necessary for hemodynamic optimization.^44^ If cardiac dysfunction occurs, dobutamine should be added.^44^ Norepinephrine should be immediately initiated, even in a peripheral access, preventing prolonged hypotension, which yields high mortality.

Extracorporeal membrane oxygenation (ECMO) might be necessary for patients with acute respiratory failure refractory to initial measures.^45 , 46^ At first, venovenous ECMO is indicated for recovery of pulmonary function.^46 , 47^ When associated with significant cardiovascular impairment in patients with severe ventricular dysfunction and/or cardiogenic shock, venoarterial ECMO might be considered.^48^ ECMO should be initiated before the installation of failure of multiple organs.^49^

**Specific treatment.** At the present time, the treatment of critically ill patients is based on supportive measures for organic dysfunctions. Since the beginning of the pandemic, an effective antiviral treatment for COVID-19 has been sought. In China and Italy, in severe cases and in an individualized manner depending on the institution, drugs like chloroquine (CQ) or HCQ, lopinavir/ritonavir, remdesivir and favipiravir have been used. Remdesivir and favipiravir are broad-spectrum antiviral agents, whose efficacy and safety for the management of patients with COVID-19 are being assessed in randomized clinical trials.^50^ A recent randomized and controlled study has shown that the lopinavir/ritonavir combination, used in the management of HIV infection, is ineffective against the SARS-CoV-2 infection.^50^

Chloroquine diphosphate and HCQ sulfate are well-known useful drugs to treat malaria and autoimmune diseases, such as rheumatoid arthritis and systemic lupus erythematosus. In experimental studies, CQ and HCQ have shown to act against SARS-CoV-2, by interfering with ACE2 glycosylation, thus reducing the efficiency of the binding between ACE2 of host cells and the coronavirus surface protein. In addition, those drugs act by increasing the pH of endosomes and lysosomes, thus preventing virus/host cell fusion and subsequent viral replication. Moreover, HCQ prevents the presentation of viral antigens to T cells and inhibits the transcription of proinflammatory genes, hindering the release of cytokines. Thus, in experimental studies, CQ and HCQ have prevented viral entry into the cell and replication, as well as attenuated the inflammatory response. In China, a study has shown that CQ was linked to a higher percentage of clinical and virological cure, being then adopted for the treatment of COVID-19 in that country. A small study has reported that HCQ, regardless of combination with azithromycin, reduced the SARS-CoV-2 RNA detection on respiratory tract swab samples, but that study has not assessed clinical outcomes.^51 - 53^

The major side effects of CQ and HCQ are gastrointestinal intolerance (nauseas and vomit) and, in the long-term use, retinopathy, maculopathy and cardiomyopathy. Other common side effects of those drugs are total atrioventricular block, bundle-branch block, cardiac arrhythmias, hypotension, *torsades de pointes* , T-wave inversion, ventricular fibrillation, and ventricular tachycardia, which are even more frequent with their prolonged use and in the presence of liver and kidney dysfunction. On March 10, 2020, the *Journal of Critical Care* has published a systematic review on the efficacy and safety of CQ for the treatment of COVID-19, including one narrative letter, expert consensus paper, one editorial, one *in-vitro* study, two national guideline documents, and the description of 23 ongoing clinical trials in China.^54^ On March 21, 2020, the president of the United States urged the FDA to quickly approve CQ and HCQ for the treatment of COVID-19. However, the FDA currently recommends the compassionate drug use until scientific evidence on the efficacy of CQ, HCQ and azithromycin for the treatment of COVID-19 is available.

On March 23, 2020, two studies conducted in Brazil were approved by the Brazilian Committee on Ethics in Research (CONEP): a) a phase IIb study to assess the efficacy and safety of CQ diphosphate in the treatment of patients hospitalized with SARS-CoV-2: a double-blind, randomized, clinical trial – a multicenter study with 440 patients proposed by the Teaching and Research Board of Fiocruz Amazonas – that has included 50 patients so far; and b) an assessment of the safety and clinical efficacy of HCQ in association with azithromycin for patients with SARS-CoV-2 pneumonia – a multicenter study with 400 patients proposed by the Brazilian Israeli Beneficent Society Albert Einstein – waiting to start recruiting.

Since March 25, 2020, the Brazilian Ministry of Health has adopted that drug as an adjuvant for the treatment exclusively of the severe forms of COVID-19, while also maintaining the other supportive measures. The indication considers that there is no other effective specific treatment available at the present time and that the recommendation can be modified at any time, depending on new evidence. On March 31, 2020, in a preprint study, without peer review, a Chinese group showed the superior efficacy of HCQ for mild pneumonia in 62 patients assessed (with a control group).^55^ That should be confirmed in a study with higher sample power and stricter methodology. Other drugs being analyzed are glucocorticoids, immunoglobulins, interferon, and tocilizumab.

**Cardiopulmonary resuscitation.** When patients with COVID-19 have a cardiorespiratory arrest, special care should be taken, with special attention to airway management, because of the higher risk of contamination of healthcare workers performing aerosol-generating procedures.^56 , 57^ All healthcare professionals in contact with patients with COVID-19 should follow the local and national orientations for infection control and use of personal protective equipment, which should be readily available.^58 , 59^

SARS-CoV-2 infected patients at risk for acute deterioration or cardiac arrest should be identified early, as should those for whom a ‘do not attempt cardiopulmonary resuscitation’ applies, and that should be based on local guidelines.^58^

Hypoxia is the most probable cause of cardiorespiratory arrest among patients with COVID-19; however, all causes should be taken into account (hypoglycemia, acidosis, coronary thrombosis). The algorithms already validated should be applied according to the identification of shockable and non-shockable rhythms.^56 , 57^ Airway should be manipulated by experienced and skilled professionals. Healthcare professionals caring for patients with COVID-19, including physicians, nurses and physical therapists, are at higher risk of infection.^60 , 61^ Aerosol-generating procedures, such as non-invasive ventilation, high-flow nasal cannula therapy, and bag-valve-mask or bag-tracheal-tube ventilation, pose a particularly high risk.^62^

Bag-valve-mask or bag-tracheal-tube ventilation should be avoided, because of its elevated risk of aerosolization and contamination of the team; moreover, that type of ventilation has not proven to be superior to the mechanical one.^56^ If bag-valve-mask ventilation is necessary, the mask should be properly sealed, which requires more than one professional. In addition, the use of filters between the mask and the bag is mandatory. For those patients, the establishment of advanced airway should be prioritized and conducted by skilled individuals.^56^ If intubation fails or is impossible, other devices should be used, such as laryngeal tube or mask, to enable closed-circuit mechanical ventilation until definite access to airway is obtained, by either tracheal intubation or cricothyroidostomy.^57^

In case of cardiorespiratory arrest of patients on mechanical ventilation, to prevent aerosol contamination from cardiopulmonary resuscitation maneuvers and ventilation, the patient should remain connected to the mechanical ventilator in a closed-circuit system, maintaining FiO_2_at 100%, asynchronous mode, and respiratory rate of 10-12 bpm ( Figure 4 ).^56^

**Figure 4:** Cardiopulmonary resuscitation of patients with COVID-19. CRA: cardiorespiratory arrest; COVID-19: Coronavirus disease 2019; PPE: personal protective equipment; ACLS: Advanced Cardiovascular Life Support; FiO_2:_fraction of inspired oxygen; RR: respiratory rate.

### Thrombosis prevention and management

The literature provides suggestive evidence that the exacerbated systemic inflammatory response present in COVID-19 causes endothelial dysfunction and increased procoagulant activity, which, in association with lower oxygen supply, might contribute to coronary plaque instability or to thrombus formation on a ruptured coronary plaque, and, thus, to plaque vulnerability.^10 , 11 , 63^ It is worth noting the importance of the differential diagnosis of obstructive coronary artery disease from type II myocardial infarction.^64^ Patients with COVID-19 can present with acute coronary syndrome due to a mismatch in myocardial oxygen supply and demand, being diagnosed with type II myocardial infarction. The cases should be analyzed individually, because a large part should be managed conservatively, considering that 7% of the patients with COVID-19 and acute coronary syndrome might have type II myocardial infarction or myocarditis.^64^

The approach to acute coronary syndrome in patients with COVID-19 should consider the availability of local resources, such as structured catheterization laboratories, coronary care unit and/or ICU beds, and adequacy of the environment to the protective measures against SARS-CoV-2.^64^ A Chinese report has suggested that thrombolysis should be the first-choice therapy for patients with COVID-19. That is a controversial recommendation, especially where primary angioplasty can be performed, respecting all the safety rules for protection of healthcare professionals and hospital environment (personal protective equipment, negative pressure room, proper cleaning).^64^

The treatment of cardiovascular complications should be based on the ideal and careful use of the therapies recommended in the guidelines. Therapy with ACEI, ARB, beta-blockers, antiplatelet agents and statins should abide by the recommendations in the guidelines, respecting the contraindications related to hemodynamic stability and presence of other organic dysfunctions.^21^

Patients with COVID-19 are at a higher risk of venous thromboembolism, because of their prolonged physical inactivity and their abnormal coagulation parameters.^4^ The use of non-pharmacological prophylaxis strategies is recommended for all in-patients with COVID-19. Pharmacological strategies should be considered, such as the use of unfractionated or low-molecular-weight heparin, taking into account the latter’s contraindications and the patient’s creatinine clearance. Venous thromboembolism should be suspected based on clinical criteria, in situations such as maintenance of high d-dimer levels and refractory hypoxemia, or in the presence of echocardiographic signs of pulmonary hypertension and right ventricular dysfunction.

### Telemedicine and cardiology

Because of the exponential viral spread, social distancing has been determined as a key-factor to reduce the speed of spread by reducing person-to-person contact. The use of information technology, thus, is mandatory as an emergency response to environmental issues or biological risks. Telehealth enables remote triage, supports the diagnosis of diseases, and ensures access to routine care during an infectious disease outbreak.^65^

In 2019, the Brazilian Medical Board published a decree defining telemedicine as the technology-mediated practice of medicine aimed at health care, education, research, prevention of diseases and injuries, and health promotion, regulating that practice. The Brazilian Society of Cardiology has issued a guideline on telemedicine applied to cardiology, also named telecardiology. Telecardiology by acting in health promotion, disease prevention, diagnosis, treatment and rehabilitation, which impact the quality of life, can be considered an important ally of the health system, be it public, supplementary or private, to promote quality integral health care. The implementation of telecardiology is important to primary and specialized health care.^66^ In cardiology, telemedicine can be useful to control risk factors, such as blood pressure and diabetes mellitus, to improve the lipid profile, to reduce weight, and to increase the success rate of smoking cessation programs.^66^

At the current stage of pandemic control, telemedicine became a useful tool, especially for patients at high risk, reducing the exposure to SARS-CoV-2 and helping control comorbidities. On March 19, 2020, the Brazilian Medical Board, in accordance with the Brazilian Ministry of Health, recognized the possibility and ethical character of telemedicine regarding teleguidance, teleconsultation, and telemonitoring.^67^

General recommendations

- Intensify the care and preventive measures against the novel coronavirus infection in the population with CVD.
- Patients with CVD should be managed according to current guidelines, ensuring the best treatment available for chronic illnesses.
- It is essential that patients with CVD maintain strict adherence to proper diet, regular sleep and physical activity, avoiding tobacco and alcohol consumption.
- It is important to update vaccines. This includes the pneumococcal vaccine, because of the increased risk of bacterial infection secondary to SARS-CoV-2, and the influenza vaccine, indicated for patients with CVD.
- Outpatient appointments as well as elective tests and procedures should be postponed if clinical discretion determines they are not essential and if not performing them neither increases the risk of events nor hinders the clinical management of an underlying CVD. Telemedicine should be used to help patient’s follow-up.
- The number of healthcare professionals taking part in ward rounds for patients should be reduced, and online discussion should be implemented.

## Conclusions

COVID-19 is potentially severe and has a high spread rate. Current data available are mainly derived from retrospective studies and should be cautiously interpreted. However, current evidence already shows the need to pay special attention to patients at risk and the importance of the proper management of cardiovascular complications, with rapid identification and implementation of adequate treatment.
